# The Italian School Psychologists’ Role: A Qualitative Study about Professional Practices and Representations

**DOI:** 10.3390/ejihpe11040084

**Published:** 2021-09-24

**Authors:** Claudia Meroni, Laura Fagnani, Emanuela Confalonieri, Davide Baventore, Veronica Velasco

**Affiliations:** 1 Ordine degli Psicologi della Lombardia, 20124 Milan, Italy; claudia.meroni8@gmail.com (C.M.); fagnanilaura.l@gmail.com (L.F.); d.baventore@opl.it (D.B.); 2 Department of Psychology, Università Cattolica del Sacro Cuore, 20123 Milan, Italy; emanuela.confalonier@unicatt.it; 3 Department of Psychology, Milano-Bicocca University, 20126 Milan, Italy

**Keywords:** school psychology, school psychologists, professional role, whole-school approach, school practices, school organisation

## Abstract

School psychologists’ relevance has been broadly affirmed. However, there is no shared definition of their professional role, and more efforts are needed to promote an organisational and whole-school approach. The present study aims to investigate practices and representations of Italian school psychologists, advance knowledge of the status and development of school psychology, and learn more about the approaches currently adopted in schools. A qualitative method was used and 11 focus groups with a total of 86 participants were carried out. Ad hoc instruments were defined. The results highlighted that school psychologists are more focused on building one-on-one relationships, whereas relationships with the organisation as a whole appear to be more difficult. However, participants reported a wide range of activities, targeted to both the individual and the organisation. Moreover, efforts to strengthen the relationships with school principals and the entire school community were described. Specific needs emerged and the necessity to better define the school psychologists’ role was reported by the participants. More efforts are needed to promote an organisational approach among Italian school psychologists and specific training should be offered.

## 1. Introduction

The link between education and health has long been recognised. Good health results in better school performance, and access to supportive educational environments determines better health outcomes [1,2]. For this reason, an intersectoral collaboration between schools and the health sector is needed to promote learning, health, and well-being among students [3]. This collaboration may consider health promotion, health care, screening, assessment, and many other activities. In this area, school-based or school-linked health services embedded in the community play a crucial role [1,4]. The World Health Organization [1,4] highlights that comprehensive school health services occupy an essential place to contribute to the health and well-being of school-age children: They can reach children directly where they spend most of their time and have direct access to their families, they can reach marginalised children with clear implications in terms of equity, and they can impact multiple determinants of health. The literature underscores the importance of some characteristics of the health services targeted to children and adolescents [1,5,6]: They must guarantee comprehensive care considering all health areas (e.g., mental health, sexual health, substance use), autonomous access and use of health services should be assured and promoted, and a strong link between health services and schools is necessary.

School psychologists can play a crucial role in this direction. They are among the most important professionals in providing effective school health services. They are expected to work closely with school staff, including teachers and principals, as well as professionals from other health and social services. In the literature, a wide range of objectives and functions connected to the health and well-being of children and adults that revolve around the school setting are reported. With specific reference to students, access to school-based mental health services and support has been shown to directly improve students’ physical and psychological safety, academic performance, and social–emotional learning [7]. School psychologists’ role also affects the entire school community, and its effects are crucial to making school health services effective. Furthermore, the pivotal role of school psychologists in creating a network that connects schools to health and social services existing in the community is a key strategy of the health-promoting school approach [8]. The relevance of school psychology has increased even more significantly during the pandemic since many needs have been further exposed and intensified. Moreover, the difficult situation caused by the emergency highlighted the need for structured, well-organised, and effective psychological and social health services that could promptly respond to the crisis [9].

Surveys carried out by recent research show that school psychologists are present in the majority of schools in some capacity. The greatest proportion of their work involves consultation with students and intervention in classes, and teachers seem to appreciate the services they provide. However, significant barriers limit the impact of their work in supporting students, teachers, and families [10]. These barriers include organisational and practical issues, such as unfavourable school psychologist-to-student ratios, collaboration with multiple schools, temporary contracts, and lack of resources. Given these conditions, school psychologists frequently respond (and are expected to respond) reactively versus proactively. In other words, the emphasis in service provision is on designing interventions to address existing problems versus the more successful approach of providing more generalised preventative services to head off potential difficulties [11].

An additional issue that school psychologists need to face is related to professional identity. To date, there is no shared definition of the actual role of school psychologists. This lack of definition also impacts the perception of the school psychologist’s role among teachers, school staff, students, and families. Psychologists are typically asked to perform only one-on-one counselling and cognitive assessments and intervene when critical situations occur. They are perceived as being external to the school; they are less known, and people are not aware of their duties and functions. For instance, many teachers exclude themselves from psychologist–pupil interactions, assuming a strict dyadic relationship between the school psychologist and the children; they have never spoken to a school psychologist, and many feel they need a better understanding of their role in providing consultations [12]. In other cases, psychologists are perceived as part of the school community; they are integrated into the school system and work towards the same goals. In those situations, the school psychologist is considered a consultant, a person they can trust and cooperate with to achieve improvements in the educational setting.

In light of this, the role of the school psychologist should be better defined. Overall, many studies on the effectiveness of school psychology agree that an organisational and consultative approach addressed to the school community as a whole would be preferable to relying on traditional approaches in which the focus is on working only with a child [13]. From this perspective, school psychologists should practice advocacy and community organisation functions to impact the school’s culture in a comprehensive manner.

In this direction, the literature has already identified a wide range of activities to be implemented and objectives to be achieved by school psychologists. A significant percentage of school psychologists in early childhood services continues to spend a substantial portion of working hours completing screenings and special education evaluations [11,14]. However, psychologists in educational institutions have a more general task of contributing to the promotion, implementation, and evaluation of the skills necessary to support the development of positive attitudes towards learning in students through the promotion of cross-cutting activities with a lifelong learning perspective [15]. The COVID-19 pandemic highlighted the increase in the need for mental health support to help adolescents learn to regulate their moods, resolve parent–child conflicts, and maintain peer relationships and social skills [9]. The pandemic also confirmed the pivotal importance of interventions to support and enhance the well-being of teachers, which is often overlooked in favour of actions directly focused on students. In fact, approaches that consider the health, well-being, and satisfaction of teachers by training and empowering them are always strategic, regardless of the pandemic, as teachers represent the most significant figure for students in school settings. Psychologists can train teachers to deliver activities and identify new ways to promote social skills and interactions among students, whether in virtual settings to reduce social isolation or in the classroom. School psychologists can also support teachers by exploring ways to encourage self-care and help reduce the risk of burnout. When teachers are supported, this will likely also increase their availability to support their students virtually [9]. In terms of health benefits, it is extremely strategic to work on the school environment, taking into consideration the school climate, organisational and physical aspects, and the relationships that exist not only within the school but also with the surrounding community and services [8,16]. Table 1 illustrates a partial list of activities that should fall into the domain of school psychology and could be categorised into four main areas according to international research in the field [7,10,17,18,19,20].

It is to be noted that the tasks displayed in Table 1 cover the entire continuum, from prevention to treatment, rehabilitation, and evaluation. Moreover, it appears clear that school psychologists should work with different targets and categories, which include but are not limited to children and adolescents.

A similar systematisation allows greater importance to be given to the area of organisational efforts that are often left behind and receive less attention. In this way, strengthening the consultative perspective of the role of school psychologists is possible. An initial analysis of the international literature on school psychology shows that significant attention is given to the actions and interventions that school psychologists carry out in specific contexts [14,21,22,23]. However, less research has focused on the complexity of the psychologist’s role and its implications for the well-being of the community as a whole, both at the micro level and in terms of macro-coordination with the educational community. Moreover, little to no attention is dedicated to psychologists’ needs and to the quality of their relationships with the stakeholders they interact with in the school system.

The situation of Italian school psychology is particularly significant and requires further investigation. Despite a large number of professionals working as psychologists in the school setting, Italy is still a country in which many aspects of the status of school psychology are critical. Some limits of the Italian conditions emerge from local analysis and from the main cross-national surveys about school psychology, such as the study promoted by the European Federation of Psychologists’ Associations (EFPA) and the Network of European Psychologists in the Educational System (NEPES) [20] or the International School Psychology Survey (ISPS) carried out by the International School Psychology Association (ISPA) [24]. The first major critical factor is the lack of laws or regulations that define school psychology professional standards or require school psychologists to be credentialed [20,24,25]. Due to this, the presence of psychological services in schools is often left to autonomous initiatives by schools themselves [26]. A wider and more structured system, present in other European countries, that includes psychologists, educational and pedagogical consultants, other professionals, and teachers that take care of the needs of the whole organisation, is lacking [27]. Moreover, as school psychology services are administered on an independent contractual basis in public schools, monitoring the presence and distribution of school psychologists in Italy is difficult. The absence of a national school psychology association and of university programs that provide doctoral-level preparation for school psychologists represent additional lacks for Italian professionals in the field [20,24,25]. Finally, although Italian welfare provides public health services embedded in the community, a better link with schools is often necessary. In these undefined contextual conditions, a precise definition of school psychologists’ professional role is lacking in Italy. Activities implemented depend on the individual initiative of schools or psychologists and the organisational and consultative approach suggested in the literature [11,13,14,15] is undervalued.

Since the onset of the COVID-19 pandemic, which hit Italy from the very first stage, much investment has been made to improve the provision of psychological support in the school setting, both in terms of public expenditure and the establishment of more structured services. Funds were allocated to schools to allow the recruitment of school psychologists to respond to the crisis. At the end of 2020, the National Psychologists Professional Order issued a set of guidelines on the promotion of psychological well-being at school in collaboration with the Italian Ministry for Education [18]. This document describes areas of interventions and dimensions that should fall under the remit of the school psychologist and should be further disseminated among practitioners, as it represents a useful tool to define and guide the position of school psychologists even once the pandemic is over.

Research evidence on the condition of Italian school psychologists is still not extensive, especially when compared to the international literature on this topic. Some studies have focused on the Italian context [23,28], and a recent study by Matteucci and Farrell [10] contributed to depicting the presence and practice of school psychologists. This study is particularly relevant because it used a mixed-methods design, but data collection focused only on one Italian school district. Moreover, the evolution of comprehensive approaches to school psychology and the development of consultative whole-school strategies in the Italian context still need to be investigated.

As Italian research on the topic is still lacking and the role of psychologists is not clearly defined or integrated in the school system, we aimed to investigate and analyse existing practices in order to identify key elements and support the definition of school psychologists’ roles and functions in the Italian context. In particular, we wanted to ascertain the extent to which a consultative and organisational approach is currently adopted by school psychologists working in the Italian context. The specific objectives were to:

- Examine psychologists’ perceptions of schools;
- Investigate and describe the activities reported by psychologists in the school setting, and categorise the topics addressed by their interventions and the tools used to support their work;
- Explore the perceptions about the relationship between school psychologists and schools community members;
- Identify the specific competences that psychologists believe to be necessary to work properly, as well as the needs and the barriers they face in their work;
- Explore the psychologists’ perceptions about the changes that have occurred in their professional role in educational contexts in Italy (both over the years and during the COVID-19 pandemic).

## 2. Materials and Methods

### 2.1. Participants

The study used a qualitative approach, and 11 focus groups were carried out in different provinces of Lombardy with a minimum of 5 and a maximum of 10 participants each. Table 2 reports the characteristics of each focus group.

Overall, 86 school psychologists participated. Participation required the following criteria: to be registered with Lombardy’s Psychologists Professional Order, to practice the profession in the Lombardy territory, and to currently be in the role of school psychologist. Most of the psychologists who took part in the study were female (10 males and 76 females), and the average age was 44.3. They were characterised by different professional experiences and roles in schools, and the length of service ranged from 1 year up to 20+ years of professional experience. They worked at all educational levels: preschool, primary, and secondary school. Table 3 shows the averages and percentages of the characteristics of the participants in the focus groups.

### 2.2. Procedure

This qualitative study was conducted in Italy between April and May 2021. Participant recruitment was carried out through online platforms, newsletters, and social networks of the Lombardy Professional Order of Psychologists. The school psychologist had to read all the information about the study, sign the informed consent form, and fill out a questionnaire to communicate personal and professional data. This form was used to screen the participants. Following verification of the requirements under study, the participants were assigned to the focus groups based on the province in which the schools they operate were located. Participation was voluntary.

The focus groups were conducted in an online mode and were audio-recorded. For each focus group, there was a conductor with experience in the field of health promotion and school psychology and a recorder. Conductors and recorders were first briefed about the instruments used and confidentiality procedures. The focus group sessions lasted around 2 h. The study was approved by the Council of the Lombardy Psychologists Professional Order of Psychologists (sitting of 14 January 2021), who reviewed the study for ethical standards. Maintenance of confidentiality was assured by the researchers and the participants were asked to maintain confidentiality, too.

### 2.3. Instruments

A questionnaire was used to collect participants’ personal and professional data and to screen them according to the participation criteria. The personal data collected included age and sex. Regarding professional expertise, the following information was requested: place of work, work experience, hours per week working as a school psychologist, and characteristics of the school (order and grade). Finally, the questionnaire asked for practical information regarding contacting the participants and scheduling the focus group day.

The conductors used a semi-structured focus group guide (see Appendix A), which was carefully designed to encourage the participants to express themselves on issues pertaining to the role of the psychologist in the school system. The focus track was divided into five different areas corresponding to the macro-objectives of the research. Each area was explored using different techniques.

The first area investigated the psychologists’ perceptions of the school where they work. Free associations and metaphors were requested and used as starting points for a group discussion. Metaphors were considered images that exemplify ideas about the school organisation as a whole and the relationships within it [29,30]. The second area explored activities, intervention topics, and tools used by psychologists during their work as school psychologists. A conceptual map was created with the group, and each activity typology was examined in depth. Then, the needs of the school, which were identified during these activities, were investigated. The third macro-area explored the relationships and networks between psychologists and the school context: the relationship the professional had with his or her contact person and how he or she established relations with students, teachers, parents, and other professionals working at the school. The fourth area of the focus group inquired about the specific competences a school psychologist is required to have and the needs and barriers they face. A word cloud about psychologists’ competences was created as a starting point for the discussion. Finally, the participants were asked how their work had changed over the years, how much this change was influenced by staying in the same school for several years, and how the COVID-19 pandemic had affected their work.

The instruments were designed ad hoc based on a careful review of the literature with similar objectives.

### 2.4. Analysis

A quantitative statistics profile analysis was performed on the data collected from the screening questionnaire. A thematic analysis of the focus group data was carried out through an inductive and “bottom-up” process [31,32]. A constructionist paradigm was followed, assuming that experiences, realities, and meanings are the effects of discourses operating within society. For this reason, data were analysed both at an explicit level, describing the semantic content, and at a latent level, interpreting meanings and implications of participants’ words and language. The free associations and the metaphors used by participants were interpreted to understand professional representations and relationships with the school. Metaphors can activate an imaginative thought that is useful for identifying the kinds of framing used to attribute meaning to their professional role and context [30,33,34,35,36,37].

A step-by-step procedure was used through a recursive process moving back and forth throughout the phases until data saturation was reached. In the first phase, the conductors autonomously analysed the transcription of the focus groups to familiarise themselves with data and generate initial codes from the entire data set. Afterwards, a debriefing moment was organised between the conductors and the study coordinator, following the study objectives. Themes and sub-themes were identified, sorting and collating the different codes and progressing from descriptive to interpretative analysis until agreement was reached. A first thematic map was defined, identifying relationships between codes and themes. In a third phase, codes and themes were reviewed and refined. Additional data that were overlooked in the first coding stage were coded within the themes. Then, an initial study report was written to describe the detailed results. In the next step, the analyses carried out and the report were presented and discussed again with all authors and coordinators of the broad project. New themes and sub-themes were identified and the thematic map was enriched. Finally, the codes and themes were reviewed and refined again according to the new inputs and the final report was written.

## 3. Results

### 3.1. Perceptions of the School

The analysis shows how school psychologists perceived the school as changeable, multifaceted, and uneven but extremely heterogeneous. It was defined as a “community in the community” because it is rich in many elements and has many involved actors who collaborate through a network. The complexity and plurality of schools were perceived as positive, valuable, and enriching. It is definitely a symbol of the plurality and importance of such organisations. On the other hand, having so many factors at play makes schools challenging and complex to interact with, as it implies responding to several different needs, opinions, and situations.

“*It’s really a community in the community. I am lucky because I don’t work alone in my school. And schools respond positively to my project that implies actions not only within the school itself but also with external family counselling services. The schools I know work exactly like that, like communities that are open to the larger local community.*”

(Focus Group 5)

“*Considering that this school really is a small town, in it you can find a lot of multi-faceted situations. For example, the teaching staff includes a number of opinions, sometimes even diametrically opposed. […] Often schools spread over multiple sites. This complexity sometimes makes the work more difficult. Besides being a community in the community, you also have to deal with this plurality.*”

(Focus Group 5)

During the group discussion, stimulated by free associations and metaphor definition, participants described the school as a variety of animals with peculiar characteristics that sketched out the complex picture of the schools they worked with.

“*A platypus! When it was discovered, they didn’t know how to classify it… it has so many separate characteristics that cannot be linked to one another; it’s a composite institution: an animal that is strongly influenced by the surrounding environment, as it needs a specific context to survive.*”

(Focus Group 3)

“*To me it is a Chimaera, the mythological animal, that has many heads, many identities… or a turtle, a centennial turtle because the school has a shell, an armour, a rigid scaffolding, and sometimes it pulls back into its shell. And sometimes the effort is being able to help it open to the outside world. It moves slowly, but it moves…*”

(Focus Group 4)

Thinking of the school in which they work, the professionals described it as a collaborative and flexible structure where people can be listened to and trust each other. It was also perceived as inclusive, welcoming, and engaging. It tries to be as attentive to and careful as possible with everyone’s needs. It manages to put in place many resources even during a crisis and is rich in potential, creative, and constantly evolving. Overall, it shows a tenacious and combative character, able to wait and push through difficulties when necessary.

Psychologists perceived the school as a reality that needs to be helped to understand what it needs to implement active strategies and shared best practices. It is extremely needy and demanding, constantly looking for strategies and tools to keep up with the times. However, it is an organisation that can be ambivalent at times: In some cases, schools perceive the psychologist as a salvific individual, whereas in other cases, they oppose and resist the psychologist’s role. Similarly, the school is open to listening to psychologists’ suggestions and recommendations, but it often becomes more defensive when additional interventions are proposed.

From a perspective that focuses on the possibility of change over time, the school was perceived as “slow”, as it must cooperate with different actors and entities. It is also often slowed down due to bureaucratic formalities. Because of this, the school is in some cases “closed” and “aggressive” to defend itself from the perception of excessive demands and tasks that come from the outside (bureaucracy, absent services, lack of tools, extremely broad mandate). This is why the great potential of schools often remains unrealised:

“*It’s a tiger in a cage: aggressive towards the outside, especially parents and other people; it’s a fighter, but at the same time it doesn’t have well defined tools and roles. It’s forced in a reality that is too tight, but at the same time, it’s closed by choice… because it’s closed to external opinions… it fights with the few tools at its disposal. It has many potential positive aspects, but it’s stuck.*”

(Focus Group 10)

“*I agree with this idea of aggressiveness and with the picture of a cage. Aggressiveness among staff members because of different opinions and visions; aggressiveness towards the outside world… there are proactive initiatives but sometimes those are stopped by the bureaucracy, by internal organisational struggles… there is a great potential that never gets to be expressed, especially in this period.*”

(Focus Group 10)

During the group discussion, participants described the main critical issues and needs of the different school actors. Fragile and needy subgroups were also identified. Students with social, relational, or economic difficulties or identified as children with learning disabilities or special needs require more attention. Particular attention should also be paid to adolescents, as they are living in an age of transition in which relational and emotional difficulties often emerge, exacerbated by improper use of technology, especially during this unprecedented pandemic crisis. Guidelines to managing reports on critical cases targeted to schools and teachers would be helpful to better support these fragile categories of students. Parents were perceived as needier, as they seem to have fewer tools, skills, and attention to recognise needs and difficulties that they may encounter while their children grow up. The school principal was often perceived as overwhelmed by a huge amount of bureaucracy. Understanding internal needs and selecting requests coming from the outside can be challenging. The efforts made to deal with bureaucracy and administrative matters can lead to a reduction in attention paid to issues related to learning and educational co-responsibility. The situation worsened during the COVID-19 pandemic, as principals had to manage distance learning, school closures, and measures to limit the spread of the virus. Finally, difficulties were observed in the management of changes in teaching. In particular, teachers need up-to-date tools and training to understand and read students’ behaviours. This would help organise experiential, active, and modern teaching techniques that place students and their needs at the centre instead of only focusing on superficial factual knowledge. Difficulties were also found in empathic and relational exchanges with students because it is hard to keep up with the world of young people, which evolves at a very fast pace. Overall, the teaching staff experiences excessive professional burdens; like the school management, the shift from face-to-face lessons to online teaching and the urgent requirements of technological skills during the pandemic intensified this overload.

### 3.2. School Psychologists’ Activities and Topics

Activities and topics addressed by the participants in the study during their work in the school context are reported and described below.

The activities carried out by Italian psychologists are extremely numerous and heterogeneous. This wide variety of activities can be interpreted based upon two factors connected to the target of each action. The first factor refers to the position of the target groups with respect to the school organisation: internal members or external actors. The second factor identified is the numerousness of the target addressed: from an individual target to a group target.

**Figure 1:**
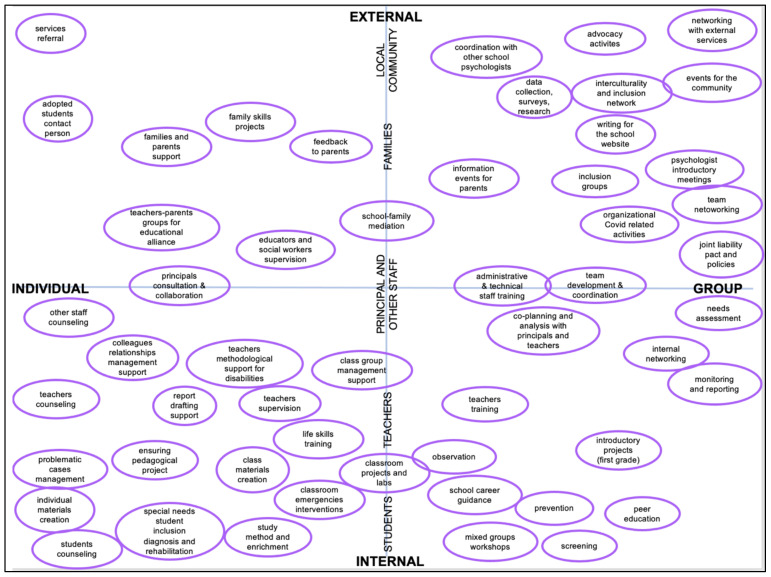
School psychologists’ activities.

Each element is represented within a scatter plot (Figure 1) according to the factors described above: The vertical axis displays the activities based on whether the target they are addressing is internal or external to the school organisation. From this perspective, activities range from students, teachers, management, and administrative and supervisory staff to interventions with families and the local community from a systemic perspective. On the horizontal axis, activities are placed on a continuum based on the dimension of the target to be reached: from the individual level to activities that affect the entire school structure.

The number of activities reported was much higher than initially expected. However, interventions that focus on the single case and the class group are still those that psychologists dedicate most of their time to. Overall, activities addressed to an individual target are still the main area of intervention. Activities addressed to groups or the whole organisation are less frequent. When group activities are delivered, they are mostly linked to networking, communication, and information. Group practices to support students are less common, and group practices aimed at the teaching staff are even more uncommon.

Consistent with this, the tools used by psychologists are mostly geared towards interventions with a single student, problem, or class. Less space is devoted to observation and needs assessment. The school context often requires targeted and specific problem-solving interventions without giving an opportunity to observational activities. However, the preliminary phase of assessment was considered important by a large number of the participants, who expressed their desire for more space and time dedicated to this activity.

In addition, interventions are still implemented in the classroom directly with students more often than expected, and there seems to be less commitment to teacher training. Training teachers who ultimately deal with children every day would be preferable to achieve a stronger impact on students’ behaviours. Moreover, few activities were reported to promote teachers’ well-being.

Families are a target that was also taken into account; however, tailored interventions specifically designed to meet the needs of a given family were mentioned only in a few cases.

Regarding the topics faced by the psychologists, the most important specific themes cited by the participants were organised into three main areas. The first area includes themes with a focus on problematic aspects and criticality, such as self-harm, fragile families, abuse, emergencies, developmental issues, and impairments. This area is the one more closely related to the idea of disease and to activities focused on the single case.

The second cluster identified considers topics related to well-being and health. It is linked to the idea of positive psychology and includes most of the topics addressed by health promotion and prevention, usually through group activities carried out both with the class or with a whole organisation. Some of the topics included in this cluster are life skills, emotion management, risky and healthy behaviours, motivation, and information and awareness.

The third and final cluster includes the topics of relationships and school inclusion. It ranges from relational difficulties to inclusion of foreign student. Relationships among school staff members and school–family relationships are also part of this group.

Among the topics addressed by participants, sexual education, social skills, sexual orientation and LGBT community, school climate and cohesion, and parenthood and life cycle also deserve to be mentioned. Those themes can be placed in the second category because of their strong connection with well-being, but they are also close to the third cluster, as some elements could partly belong to inclusion/relational areas.

Themes specifically related to the response to COVID-19 were not considered in this research. However, topics such as isolation and loneliness were reported by participants as an existing issue to be addressed regardless of the pandemic that contributed to their exacerbation. The same applies to digitalisation and new media. Those themes, together with communication in general, can be considered cross-cutting topics pertaining to all three identified areas depending on how they are classified.

Interventions carried out by participants when working in schools are mainly attributable to the area of problematic issues and to the area of well-being and health; slightly less attention seems to be dedicated to inclusion and relationships. The psychologist is often seen as a problem solver, and, unfortunately, in many cases, is not yet seen as a prevention professional. When psychologists offer preventive projects, they are mostly addressed to the class group and cover a wide range of topics related to health promotion (bullying, affectivity and sexuality, risky behaviours, emotions, anxiety management, etc.). Moreover, less attention is dedicated to learning issues.

### 3.3. Relationships and Networks between Psychologists and the School Context

Psychologists reported a positive relationship with the school with which they collaborate, characterised by a climate of empathy and cooperation with the institution. However, the work at school requires them to be constantly engaged in a number of relationships with different school actors and stakeholders.

Broadly speaking, they are very well known by the students and by the teachers who strongly believe in their work. However, the connection is weaker with the rest of the teaching staff and with students’ families, who are, in many cases, mostly passive recipients of feedback reports, training, or themed events.

The relationship with the school principal or teachers with managing roles (or their representatives) is crucial for health professionals working in schools. Participants affirmed that the relationship with the principal is essential to access the organisation and to work effectively as psychologists. If the principal is farsighted and engaged, it is possible to build a common vision working towards the same goals; in contrast, if principals are perceived as less cooperative or confused, the collaboration is fragmented.

“*Organisation and participation go together; those are the things that allowed me to work better in some schools rather than others. I’ve truly seen the difference, in particular when dealing with the principal. The difference between an organised, cohesive management that works well together, with whom you can exchange views very openly because they are ready to respond, they are there for you, they follow you and help you build the process. And on the other side disorganised principals, who are a bit lost, may be because they are overwhelmed by bureaucracy, by continuous changes and relocations… who were not able to build a more coherent process with us, with me, because they were continuously shaken by various internal organisational problems. Therefore, when the school was well organised, the collaboration has always been good, smooth. While when roles on who does what were not clear, it was a bit more difficult.*”

(Focus Group 5)

However, the results show that collaboration with school principals is functional only in the early stages of the working process and during the needs-assessment phase. Principals seem to be less involved in the actual planning of interventions or in daily activity implementation and monitoring. As a consequence, good outcomes of the project depend almost exclusively on the psychologist and on teachers’ support. On the other hand, psychologists did not mention the possibility of delivering management consulting activities to support principals.

Overall, school psychologists’ relationships with students, families, school staff, and principals are influenced by several factors. In general terms, their role is better known at lower school grades than in high schools, also due to the dimension of the school itself. The type of interventions they implement also impacts the development of their position in the school context. If projects are addressed only to students, their role is less acknowledged; if they also cooperate with teachers, they succeed in being more integrated into the school community.

Of course, seniority, length of service, and continuity in the same school are also key elements to developing strong interactions, as a consultative approach has to draw on continuity over the years. Likewise, the number of institutes in which a psychologist operates at the same time can impact the quality of relationships because building fruitful alliances requires focus and energy. Moreover, when teachers and principals agree on the importance of health promotion at school, the development of good relationships and favourable working environments is easier.

The focus group results confirmed that the participants are aware of the importance of establishing a strong relationship with the different actors in the school community. This is why all psychologists organise introductory meetings and presentations when starting at a new school and at the beginning of every school year. The way in which the psychologists introduce themselves to the school context can result in different responses. Visiting each class and getting to know people face to face while explaining the services that will be offered is usually more effective than online video presentations or large meetings open to everyone. After the introductory phase, the school psychologists said they believe a period of observation is beneficial to reduce the distance and initial distrust school members feel at first with an external figure.

Regarding relationships with relevant services that exist in the community outside the school context, participants generally mentioned good collaboration with local services such as social services, family counselling, and hospital child neuropsychiatry. For referral and joint projects, they also have a constructive relationship with a number of professionals from the private sector, including lawyers, speech therapists and psychomotor therapy specialists. However, when asked about networking with other services, participants did not make references to local authorities. Social cooperatives and public health services in charge of local policy coordination and health promotion were only mentioned in a few cases.

Overall, strong network connections (both internal and external with respect to the school) are essential to be able to work effectively: In some cases, the network already exists, and the school psychologist integrates into it when starting at a new school. In other cases, the psychologist is requested, explicitly or implicitly, to build such a network as part of his or her mission. The existence of a well-established network between the school and local services can facilitate the integration of psychosocial professionals into the school context from the very beginning: Building a network from the ground up is far more demanding than enhancing an existing one.

“*…team-work, exchange of views with colleagues… If collaboration and cooperation are possible then you can achieve something. Otherwise, if the school has a character that delegates to the specialist… you know that some realities work that way, you can try to change them but only to a certain point, exactly because the psychologist is missing. It’s the person that should be in charge of the needs assessment, of listening and following the school, taking care of the school over the years and not only for brief projects*.”

(Focus Group 10)

### 3.4. Psychologists’ Competences, Needs, and Barriers

Consistent with the considerations above, participants recognised and described the ability to listen and empathy as two of the most important basic skills to work as psychologists in the school world. Flexibility was also seen as essential to manage diverse situations and build relationships of mutual trust.

“*…creating a human relationship with teachers is important, and being flexible, being able to listen and to look at the context’s specific needs. You also need to be able to use specific tools…like assessment tests… and a cross-cutting training that is focused on the target of people we will work with… for example training on students’ life cycle, specific interventions that are feasible based on the age and on the life cycle phase.*”

(Focus Group 3)

“*you need to recognise your limits. Which specific activity can the psychologist deliver? What are we allowed to do and what can we not based on the context? It’s not the right setting for psychotherapy.*”

(Focus Group 3)

“*I believe creativity is an important skill: sometimes keep thinking about what you could do if you had more time is useless… You have to adapt to what is available, take advantage of creativity to get the chance of a project taking advantage of actual the resources at your disposal.*”

(Focus Group 10)

Considering the numerous competences mentioned by participants, it is possible to operate a subdivision based on the specific type and features. Through a content analysis, four macro-categories can be identified. The first category refers to cross-cutting competencies that broadly apply to the whole professional experience when dealing with the school organisation. It includes skills such as flexibility, autonomy, dealing with complexity, and multitasking that participants believed should be developed to address school complexity. The second group of competences is that of needs assessment and observation skills. The ability to listen was the most frequently mentioned competence. Knowledge of the context, understanding of the school system, and needs and demand analysis are also part of this category. The third category is the one linked to intervention skills, which allow effective implementation of the expected activities. For example, participants mentioned problem-solving, experience, and planning. The last category identified is the one specifically focused on relational skills. “Empathy” is the word that occurred most often, followed by a number of skills considered essential to maintaining optimal relationships with the school community: mediation, understanding how to create a human relationship, networking, etc.

When asked about the barriers they face and about what would allow them to work more effectively with schools, participants described barriers that can be ascribed to the organisational context and professional and training needs.

With respect to the first category, some contractual aspects emerged as something to be addressed. A higher number of working hours and a more favourable school psychologist-to-student ratio would be critical to be able to meet all school needs. The topic of continuity emerged as crucial from the focus groups: Longer contracts in the same school that do not finish at the end of the school year would be necessary to build productive relationships and avoid interruptions that are perceived as closures and that result in difficulties in managing constant change, both for students and teachers. Additionally, the presence in the same educational institute of more than one psychologist with different tasks and objectives was also considered helpful by some participants.

“*To have more time available, for those who follow several institutes the hours are 4 or 5 for each school, to create better relationships more time is needed. [....] Need time and stabilisation, to create relationships, make the network effective, monitor situations and implement interventions. Also important to have a medium-term vision.*”

(Focus group 6)

Some practical problems can also be included among the barriers in the organisational context. Psychologists reported experiencing some difficulties when referrals to public social and health services are needed, for example, when they request the intervention of child protection services. More generally, specific situations in which a good network with local administrations and authorities is helpful can be experienced. For example, the participants mentioned that they would like to have more support from external services and believed the role of the school principal could be helpful to improve the collaboration between schools and external services.

Furthermore, the psychologists reported difficulties managing collaboration among different professionals involved in taking charge of critical cases or situations. Since different professionals and institutions follow different protocols, an agreement on the procedures to address every single case might be needed.

On a deontological level, collecting informed consent from both parents when working with children is a requirement established by the Italian psychologists’ code of ethics, even when delivering preventive projects to the whole class. In some cases, this is perceived as difficult and time-consuming. The organisational context also poses concrete barriers due to the lack of appropriate spaces, rooms, or offices for health workers inside educational facilities.

Regarding the category connected to professional and training needs, the most important element to be addressed is the need for guidelines and a definition of the role of school psychologists that participants believed to be lacking, thus producing poor homogeneity in interventions. Specific regulations, ministerial recognition, and validation from the school system would strengthen the professional role.

The focus groups also highlighted, in terms of professional and training needs, the wish to have networking opportunities with health workers and professionals from other sectors. Having access to continuous training and development courses with other colleagues would also be welcome. Training topics should address specific topics related to education and should expand the knowledge and availability of a wider range of tests and assessment tools. The psychologists also felt they would benefit from a better understanding of the school context and from being closer to students’ experiences and lexicon.

### 3.5. Perceptions about School Psychologists’ Role Changes

During the focus groups, the participants’ perceptions about the changes in the role of school psychologists were investigated. Participants reported that a process of acknowledgement and integration of the position is currently underway in the school system. In their opinion, some important steps have been made in the last few years, even if much still needs to be achieved in terms of professional recognition. First, from their point of view, the role of psychologists in schools is now seen as more important than previously; this seems to be the case at all educational levels, including nursery schools. Moreover, people are beginning to understand that the school psychologist is not a professional to be consulted occasionally but someone to rely on for a complex process characterised by a high level of continuity.

“*I’ve been lucky enough to work at the same school for several years and I’ve noticed an evolution in the level of trust, recognition of myself and my role. Initially I was looked at with a certain detachment, but now that I’m in my third year there is an atmosphere of cooperation generally and I feel I’m in a familiar environment.*”

(Focus group 8)

From the participants’ perspective, schools show a growing awareness that psychologists are not there to intervene in a single case or in case of an emergency only but rather to have a presence in the entire organisation through projects, prevention, and supervision. Of course, a certain degree of instability is still present: Improvements can be seen, but building a proper culture of prevention takes time. In addition, the participants underlined in the majority of the focus groups that the degree of recognition of their role by school members is very much dependent on the length of service within the same school.

In general, the participants reported fewer prejudices and biases in accessing psychological services by students, who see it as an opportunity to grow. In contrast, they thought that teachers still find it hard to acknowledge the role of psychologists, to the point of substituting for them on some occasions. This occurs, although the psychologists perceived teachers as being in extreme need of tools and strategies to understand students and keep up with the times that are changing at a faster pace than in the past.

With reference to collaboration with social and health services outside the school, the psychologists believed it has increased over time, making the building of a cooperative network easier.

The participants also pointed out changes over time connected to contractual aspects. In the past, they could count on more funding from local authorities and municipalities, whereas today, they are mostly recruited directly by schools. This consideration has practical implications for their work: They do not receive economic compensation for tasks carried out outside the school hours (e.g., team meetings), and having different employers implies slightly different objectives because schools’ and local authorities’ requests are not always the same.

In the opinion of the participants, the COVID-19 pandemic provoked additional changes in their daily work, with both negative and positive consequences. Some participants thought that the greater schedule flexibility caused by online consultations made access to psychological services easier, whereas others struggled more to reach children and adolescents through a monitor since distance makes relationships more fragile. Many resources and energy that were inconceivable before the crisis emerged in recent months, but the psychologists also had to deal with frustration and suffering, social isolation, and distress among students and teachers.

Participants reported very different experiences with students’ parents during the pandemic: In some cases, the network with the families was completely lost, whereas in other school contexts, online contacts significantly increased the frequency of contacts.

Opposite outcomes were also reported in relation to changes in the perception of school psychology; some participants stated that COVID-19 helped reduce the stigma of counselling, leading many more students and teachers to ask for psychological support. They also thought that despite the emergency, the requests for intervention still focus on the promotion of networks and relationships. In contrast, some psychologists experienced the resurgence of old biases and prejudices, as COVID-19 brought back an idea of psychological support that is viewed in terms of problems and pathologies.

In all the focus groups, the participants agreed that the pandemic generated new problems that schools are not ready to handle and that are still not known or defined enough to be approached effectively, including an increase in dropout rates and new dangers connected to isolation and to the abuse of IT technologies.

## 4. Discussion

This qualitative research aimed to investigate the situation of school psychology in the Italian context, understanding psychologists’ perceptions, the approaches currently adopted, and the development and definition of the professional role. Generally, the focus groups highlighted that psychologists in Lombardy are more focused on building one-on-one relationships, whereas relationships with the organisation as a whole appear to be more difficult. However, participants reported a wide range of activities targeted at both the individual and the organisation. Moreover, school psychologists mentioned that they are committed to strengthening the relationships with school principals and the entire school community. Specific needs emerged, and in particular, the necessity to better define the school psychologists’ role was reported by the participants.

Going more in depth, the results highlighted both the strengths and weaknesses of Lombardy’s school psychologists’ work in schools.

The psychologists recognised the complexity and richness of schools and their capacity to change according to needs. On the other hand, schools were considered challenging and complex contexts. Many needs were recognised and ambivalent relationships were reported. Schools often perceived the psychologist as a salvific individual, but they became more defensive when additional interventions were proposed. These results confirmed that the psychologists’ professional role representations are anchored to the management of emergencies and existing problems, whereas preventive and organisational approaches are less recognised [11,12].

With reference to specific activities carried out by school psychologists in school settings, the present research highlighted some positive elements. They include the fact that Lombardy’s psychologists perform a wide range of activities, addressed to both the individual and to the whole organisation. Comparing the focus group results with the table of activities recommended by the literature included in the introduction [7,10,17,18,19,20], the participants reported all activities suggested by research in this area. Moreover, the psychologists’ activities focused mainly on critical/problematic aspects and on the promotion of health and well-being, followed by relational themes. This wide perspective is definitely a step forward with respect to a limited idea of psychology that only addresses individual mental health problems. On the other hand, the results showed that a higher percentage of working hours is still devoted to supporting students and overlooking other school stakeholders. Little attention is given to the well-being of teachers, who seem to be considered only in relation to their work with students. Moreover, the school-family relationship is often ignored or limited to minimal support, despite its pivotal importance in the development of students. The lack of individual activities expressly directed to families poses some questions concerning equity issues. Selective and indicated prevention does not seem to be included among the priorities, although it should be a primary concern for a comprehensive approach. According to the research, needs assessment and monitoring are key activities as well, and participants agreed on their importance for developing an effective consultative and organisational approach. However, time constraints and requests for intervention by the school do not allow psychologists to devote enough attention to monitoring school members’ well-being and the school climate, performing a proper needs assessment, or collecting and analysing data. Another critical matter that emerged from these results is that very little consideration seems to be dedicated to certain major topics specifically linked to school and learning outcomes, such as school inclusion and school non-attendance, as well as to dropouts. These deserve additional attention, as greater school achievements prevent dropouts and have a reciprocal positive impact on students’ well-being. Similarly, recommendations for school psychology practices underline the importance of fostering and promoting students’ and teachers’ motivation, which is something that focus group participants did not seem to consider a leading topic. Overall, the study confirmed that more time and tools are essential to achieve the fulfilment of a proper consultative approach, which is considered the most effective by current international literature [13].

The results showed good collaboration and positive relationships between schools and psychologists. The fact that all the psychologists are in contact with school management or with school representatives in charge of planning activities with them is a good starting point. The relationship with the teaching staff can also be considered quite positive, especially with teachers who directly work with them. Participants were also aware of the importance of a strong network, both internally and with external services and authorities. However, it should be noted that defensive dynamics and the lack of trust towards psychologists’ intervention sometimes interfere with a smooth collaboration. Psychologists are still considered primarily a solution for emergencies, not a requirement for more complex, ongoing support. Different beliefs and perceptions on what the school actually needs can make the implementation more difficult.

Although school principals are almost always involved in the initial phases of the collaboration, they are usually not engaged in monitoring or in the practical implementation. Psychologists could be more proactive in engaging and supporting principals through management consulting opportunities. This could determine a more fruitful relationship in the long run and enable principals to contribute to the implementation phase. The same applies to teachers who are not directly responsible for a certain project. Moreover, parents and families appear not to be involved or to know very little about psychologists’ presence in schools.

Regarding psychologists’ needs, the first key outcome that must be highlighted is that psychologists themselves believe that improving the definition of their professional role is a priority that needs to be addressed. In fact, role definition is a topic that stood out as crucial for all participants. Significant improvements have been noted in the last few years, but much still needs to be done to solve issues and difficulties connected to the professional role. Exchange of views, support, and clear guidelines are needed. This confirms that the considerations already highlighted by international research on the topic of professional role also apply to the Italian context. As stated by Farrell [17], many factors would be crucial in helping to define and publicise the distinctive nature of school psychology practice: defining criteria, setting standards, promoting links with local and national governments, producing high-quality research, and raising the profile of school psychology work by the authorities. From this perspective, professional associations at the regional and national levels could play a role in supporting a proper definition of school psychology [17]. This study has proven that this is true for the Italian context as well. Moreover, participants recognise the relevance of observational, relational, and flexibility skills that are required for a consultative and organisational approach. Continuity is a key element that could help build a more comprehensive vision of the school as a whole and in all its organisational aspects; however, a critical lack of contractual continuity over time emerged from the study. Continuity also plays a role in the definition of the role of school psychologists. This research highlighted that specific support would be useful to improve school psychologists’ work and help reduce risks. Notably, indications to make use of available guidelines for school psychology practise would facilitate the management of multiple responsibilities and help define the professional role. Moreover, specific recommendations on how to manage critical cases and informed consent following the code of professional ethics should be developed. Psychologists would also welcome and feel supported by formal and informal opportunities to exchange views and share good practices with colleagues. This support can also be useful to prevent psychologists’ work stress and burnout. According to the literature in the field, school psychologists are at high risk for developing work burnout due to their multiple responsibilities, work overload, and engagement in dealing with students, families, and other professionals [38]. Moreover, as the pandemic had a huge impact on everyday activities, support in understanding new needs exposed by the crisis and in acquiring new tools and strategies to tackle them should be provided.

Finally, the study investigated participants’ perceptions about school psychologists’ role changes. Participants reported an improvement in the acknowledgment of school psychologists by principals and teachers. Schools show less prejudice and more awareness of the different activities that can be offered by psychologists. The collaboration with social and health services is also increasing.

### 4.1. Strengths and Limitations

The study presents some limitations. First, the research was limited to a single Italian region. However, the study involved a large sample of school psychologists from different provinces and districts, and with professional expertise in all school levels. Second, the present study aggregated results from preschools, primary schools, and high schools to obtain a global picture of the condition of school psychology. This choice does not allow the school grade specificities to be taken into account. However, the organisational and whole-school approach can be applied at all school levels. Moreover, in Italy, school institutions include several school grades and the same principal is in charge of many schools. Finally, an online methodology was used because the research was carried out during the COVID-19 pandemic. In online meetings, nonverbal communication is less visible, and the group’s intimacy may be reduced, thus limiting group dynamics and inputs. However, research has shown that most of the interpersonal processes and dynamics of face-to-face interactions also characterise online interactions [39]. Moreover, online focus groups have been shown to be comparatively more informal and encourage participation more than face-to-face focus groups [40,41].

Some strengths can also be identified. The qualitative method used provided in-depth data about school psychologists’ perceptions, approaches, and definitions of the professional role. The sample was large and varied, with a good representation of the Italian psychologists. Finally, the methodology used to analyse the data followed several steps through a recursive process and involving different researchers with expertise in this area.

### 4.2. Research and Practical Implications

Further research is needed to collect data from a larger sample of subjects to confirm and extend the findings through quantitative methods. Larger-scale studies that encompass all Italian regions or are applied at the international level would offer the opportunity for additional insights. However, school system and psychosocial service organisation and distribution differ significantly among countries, which may lead to unclear results and comparisons. Moreover, additional analysis to investigate the differences between different school grades and levels would be of interest. More research is needed to define school psychologists’ role and the conditions that can promote a whole-school and organisational approach. Comparing role perceptions of school psychologists from different countries based on the specific structural frameworks that characterise each country would also be useful. For example, it would be interesting to understand whether role perceptions change and are influenced by different legislative framework and position requirements [42].

This study also has some practical implications. First of all, the results show the need to define Italian school psychologists’ role precisely. This definition was requested by psychologists themselves. Moreover, it could support a major recognition of the contribution that psychologists can bring to the school, and it is an essential condition to regulate this profession. Examples from other countries can be considered. In the research carried out by ISPA in the 192 member states of the United Nations, five evidence indicators of the development of school psychology were defined and evidence of all five was available for only 10 of the member states [24]. However, it is also important to consider that Italian welfare provides public health services embedded in the community. That requires a different organisation and role for school psychology.

Training opportunities addressed to school psychologists should be considered to meet the professional needs that emerge from this study. If trained to function in a broader role, psychologists will be able to address cognitive and noncognitive outcomes [43] in ways that reflect a holistic understanding of the context, according to which schools can produce not only knowledgeable students but also well-adjusted, empowered, and healthy citizens. Furthermore, they will be involved in schools in ways that reflect the values of the larger community [11], adopting a consultative approach that focuses on the whole organisation and its social and physical environment. Analysing the findings linked to psychologists’ needs, it became apparent that training efforts should focus on the following issues:

- Giving value to the relationship that exists between health and learning;
- Ensuring that schools acknowledge the importance of needs assessment and monitoring activities;
- Learn specific tools and strategies to implement needs assessment and monitoring activities effectively;
- Facilitating the relationship with school management;
- Acknowledging the importance of teachers’ well-being and learning strategies to promote it effectively;
- Implementing evidence-based methods to foster teachers’ educational role to promote students’ well-being;
- Developing strategies to promote organisational change;
- Acquiring tools to improve interventions in the school–family relationship.

## 5. Conclusions

The study investigated the extent to which a consultative and organisational approach is currently adopted by school psychologists working in the Italian context. In particular, it explored Italian school psychologists’ perceptions, the approaches currently adopted, and the development and definition of the professional role. The results allowed us to identify the strengths and criticalities of school psychology in Italy. Research and practical implications in this area were suggested.

## Figures and Tables

**Table 1:** Activities of school psychology identified in the literature.

**Organisational Support to the School**
Collecting, analysing, and interpreting school-level data to improve the availability and effectiveness of mental services
Providing risks and needs assessments
Monitoring of the “organisational climate”
Organisational support to the school institution: management of internal and external communications and decision-making
Coordinating with community service providers and integrating intensive interventions into the schooling process
**Support to School Staff**
Providing staff development related to positive discipline, behaviour, motivation, and mental health (including mental health first aid and psychological well-being)
Prevention and intervention to promote students’ well-being
Supporting teachers and school staff through consultation and collaboration
Support educational evaluation and experimentation
**Support to Students**
Monitoring students’ psychological well-being, needs, and difficulties through interviews, tests, and questionnaires
Designing and implementing interventions to meet the behavioural and mental health needs of students: providing individual and group counselling
Promotion of effective inclusion
Specific intervention focused on learning outcomes, educational achievements, and motivation
**Support to Families**
School–family collaboration to promote the adjustment of children and prevent dropouts
Opportunities to enhance school–family communication
Parents counselling and support

**Table 2:** Focus group characteristics.

Focus Group	Province	Time (hh:mm)	N Participants
1	Varese	01:47	7
2	Bergamo	01:50	6
3	Pavia-Lodi	02:38	5
4	Lecco-Sondrio	01:48	5
5	Brescia	01:44	11
6	Como	01:51	7
7	Milan	01:45	10
8	Milan	01:55	10
9	Milan	02:27	10
10	Mantua-Cremona	01:42	6
11	Monza-Brianza	01:50	9
Total			86

**Table 3:** Participant characteristics.

Variable	Category	Frequency	Mean
Age	25–35	18.6%	44.3
	36–45	44.2%	
	46–55	24.4%	
	>55	12.8%	
Grade of the schools in which they work	Pre-school	44.2%	-
	Primary school	65.1%	
	Middle school	64.0%	
	High school	41.9%	
Years of work experience at school	≤2	17.4%	6.7
	3–5	20.9%	
	6–9	16.3%	
	≥10	45.4%	
Work hours at school per week	≤5	37.2%	10.3
	6–10	27.9%	
	11–20	25.6%	
	21–30	9.3%	

## Data Availability

The data presented in this study are available on request from the corresponding author. The data are not publicly available due to privacy.

## Appendix A

**Table A1:** Focus group guide.

Topic	Probes
Relationship with the school	Thinking about the school you work with, what is the first word that comes to your mind? The school I work with is/is not/is always/is never… What if the school you work with were an animal? Try to describe it (look at it, the relationship you have with it, which emotions it arouses in you, how you interact with it…).
Activities and needs	Which activities do you carry out in schools as professional psychologists? (Who are the target groups of the interventions, what tools do you use: interviews, tests, observation, etc.?) What do you think are the needs of the school (teachers/students/staff/manager/organisation/class groups, families)? Which do you think are the neediest groups? Do the most vulnerable participate in the activities you propose?
Relationships	How many people at school know who you are and the activities you do? Who is your contact person at school? With whom do you decide what to do? Who do you contact if there is a problem? Who asks you to do activities? Do you also have contacts with other professionals working with the school (local authorities, services, other consultants, etc.)?
Skills and needs of psychologists	What specific skills do you think are needed to work as a school psychologist? Imagine having to prepare your own toolbox; what would you put in it? How well do you feel you are able to respond to the needs expressed by the school? Which barriers and challenges does schoolwork pose? What would you need to make your work more effective? Are you confronted with anyone, inside or outside the school, about the work you do in school? With whom?
Changes over time	If you have been working in schools for many years, how have you seen the relationship of the psychologist with the school change? How has it changed with the advent of COVID-19?
